# Standardizing Health Outcomes for Lung Cancer. Adaptation of the International Consortium for Health Outcomes Measurement Set to the Spanish Setting

**DOI:** 10.3389/fonc.2020.01645

**Published:** 2020-09-02

**Authors:** Vicente Escudero-Vilaplana, Antonio Calles, Roberto Collado-Borrell, María Belén Marzal-Alfaro, Carlos Polanco, Carmen Garrido, Jorge Suarez, Aurora Ortiz, Marilena Appierto, Marta Comellas, Luis Lizán

**Affiliations:** ^1^Hospital Pharmacy, Hospital General Universitario Gregorio Marañón, Instituto de Investigación Sanitaria Gregorio Marañón, Madrid, Spain; ^2^Medical Oncology, Hospital General Universitario Gregorio Marañón, Madrid, Spain; ^3^Bristol-Myers Squibb, Madrid, Spain; ^4^Outcomes'10, Castellón de la plana, Spain; ^5^Medicine Department, Universitat Jaume I, Castellón de la plana, Spain

**Keywords:** lung cancer, patient-centered care, outcome measurement, patient-reported outcomes, patient centricity, quality of life, standard set, ICHOM

## Abstract

**Purpose:** Lung cancer (LC) and its treatment impose a significant burden on patients' life. However, patient-centered outcomes are rarely collected during patient follow-up. Filling this gap, the International Consortium for Health Outcomes Measurement (ICHOM) developed a standard set of variables for newly diagnosed LC patients. In order to facilitate the use of this standard set, the project aims to adapt it to the Spanish setting.

**Methods:** The variables (instrument and periodicity) to be included in Spanish standard set were selected through consensus during 4 nominal groups (13 oncologists, 14 hospital pharmacists, 4 hospital managers and 3 LC patients), under the supervision of a Scientific Committee (1 oncologist, 3 hospital pharmacists, 2 LC patients advocates).

**Results:** The variables agreed upon included: (1) case-mix: demographic [age, sex, education and social-family support], clinical [weight loss, smoking status, comorbidities (Charlson index), pulmonary function (FEV-1)], tumor [histology, clinical, and pathological stage (TNM), EGFR, ALK, ROS-1, PD-L1] and treatment factors [intent and completion] and (2) outcomes: degree of health [performance status (ECOG) and quality-of-life (EQ-5D, LCSS)], survival [overall survival and cause of death], quality of death [place of death, end-of-life care and palliative care, death aligned with living will], treatment complications, and others [date of diagnosis and treatment initiation, productivity loss (sick leave)].

**Conclusion:** The adaptation of ICHOM standard set to the Spanish setting pave the way to standardize the collection of variables in LC.

## Introduction

In Spain, 28,645 new cases of lung cancer (LC) are diagnosed each year, representing the leading cause of cancer-related mortality (1). LC shows a heterogeneous histology and genetic profile, with non-small-cell LC (NSCLC) being the most frequent (2). The management of the disease is challenging and the choice of the optimal treatment is based on several factors, including the histology, tumor stage at diagnosis and patient's comorbidities and performance status (3, 4).

Despite the significant advances in LC management over the last decade, the disease still impose a significant psychological, emotional, and financial burden on patients (5–7). Disease symptoms such as fatigue, loss of appetite, shortness of breath, cough and pain have been identified as significant predictors of poor health related quality of life (HRQoL) (8, 9). Of note, systematic weekly collection of LC associated symptoms has been related to higher survival (10–13), better HRQoL and a better allocation of health resources (12).

Despite PROs relevance, their systematic collection using standardized and validated instruments is mostly limited to the clinical research setting. Standardization of health outcomes measurements, both clinical and PROs, in routine clinical practice is key to ensure an effective and efficient healthcare provision. In fact, experience gained from other fields shows that the systematic and standardized collection of outcomes is the sine qua non to improve the quality of any process (14).

The International Consortium for Health Outcomes Measurements (ICHOM) (15) is a non-profit organization that has carried out several initiatives to attain this required standardization. All standard sets developed by ICHOM share hallmark features: (a) outcomes variables are defined according to the pathology; (b) they represent the minimum set of relevant outcomes from both healthcare professional and patient perspective; (c) patients are involved in the standard set development; (d) they include PROs; (e) their methods of measurement and measurement frequency are well-defined to ensure benchmarking.

A standard set of patient-centered outcomes for LC (16) was designed by ICHOM for all patients with newly diagnosed LC, including NSCLC and small cell LC (SCLC), independently of the treatment received (surgery, radiotherapy, chemotherapy, targeted therapy and immunotherapy, palliative care). The set consists of two categories of variable: case-mix and outcomes. The former includes baseline sociodemographic, clinical and tumor-related variables. The latter reflects aspects related to the degree of health (including HRQoL), survival, quality of death, treatment complications as well as others like time from diagnosis to treatment.

The long-term goal of ICHOM initiatives is to develop a series of standard sets of variables to promote the consistency in data collection among different institutions within the same country or different countries. However, when implementing such standard sets, different aspects need to be considered: (1) doctors and patients should be familiarized with the variables included in the set; (2) the technology and instruments needed to measure the selected variables should be available; (3) the target population should be defined according to each country's clinical needs. In this light, the main objective of the present study was to adapt the ICHOM standard set of health outcomes for patients with LC in the Spanish setting.

## Materials and Methods

A scientific committee, consisting of an oncologist specialized in LC (AC), three hospital pharmacists (VE-V, BM-A, RC-B.) and two patient advocates (BG, BB), led and coordinated the project.

The project comprised four phases: (1) literature review; (2) first scientific committee meeting; (3) four nominal groups; (4) second scientific committee meeting.

### Literature Review

An update of the literature search conducted by ICHOM (16) was carried out in Medline/PubMed. The objective of the literature review was to identify health outcomes (clinical and PROs), the instruments to measure them, as well as, the frequency of measurement to be used during LC patients' follow-up. Clinical trials or systematic reviews in English and/or Spanish published between 01/01/2015 and 31/12/2017 were included in the review. Search terms and strategy are shown in Supplementary Table 1.

### First Scientific Committee Meeting

A group discussion was held with the members of the scientific committee to define the variables to be presented and evaluated in the nominal groups. According to this purpose, all variables included in the ICHOM standard set (Table 1) as well as those identified through the updated literature review (Table 2) were screened by the scientific committee.

**Table 1:** ICHOM standard set (16).

**Case mix variables**				
**Demographic factors**	**Baseline clinical factors**	**Baseline tumor factors**	**Treatment factors**	
- Age	-Weight loss	-Histology	-Treatment intent	
- Sex	- Smoking status - (never-smoker, ex-smoker, current smoker)	-Clinical stage	-Completed treatment	
- Ethnicity	-Comorbidities (modified SCQ)	- Pathological stage		
- Educational level	-Patient-reported health status (EORTC QLC-C30 and EORTC QLC-LC13)	-EGFR mutation		
	- Performance status (ECOG)	- ALK translocation		
	- Pulmonary function (FEV_1_)			
**Outcomes variables**				
**Degree of health**	**Survival**	**Quality of death**	**Acute complications of treatment**	**Other**
- Performance status (ECOG)	-Cause of death	-Place of death	- Major surgical complications	- Time from diagnosis to treatment
- Global health status (social functioning, physical functioning, emotional functioning cognitive function) (EORTC QLC-C30)	- Overall survival	- Duration of time spent in hospital at the end of life (last 30 days)	- Major radiation/systemic therapy complications (CTCAE^TM^)	
- Fatigue and pain (EORTC QLC-C30 and EORTC QLC-LC13)	-Treatment-related mortality			
- Dyspnea and cough (EORTC QLC-LC13)				

*SCQ, Self-administered Comorbidity Questionnaire; EORTC, European Organization for Research and Treatment of Cancer; QLC-C30, core quality of life questionnaire; LC13, lung cancer-specific quality of life questionnaire; ECOG, Eastern Cooperative Oncology Group; FEV1, forced expiratory volume; ALK, Anaplastic Lymphoma Kinase; EGFR, Epidermal growth Factor Receptor; CTCAE, Common Terminology Criteria for Adverse Events*.

**Table 2:** Variables and instruments identified in the literature review.

**Case mix variables**		
**Demographic factors**	**Baseline clinical factors**	**Baseline tumor factors**
- Employment status - Children at home	- Number of organs involved	- PD-L1 expression - ERCC1 and RRM1 expression - CEA/CYFRA 21-1 biomarker signature determination
**Outcomes variables**		
**Survival**	**Disease control**	**Quality of death**
- Disease free survival - Time to progression - Time to recurrence - Disease progression - Disease recurrence	- Tumor response - Tumor reduction	- Aggressiveness of health care
**Measuring instruments**		
- CCI: to assess comorbidities - SF-36 questionnaire: to assess health related quality of life - HADS: to assess anxiety and depression. - MSAS-SF: to measure the frequency, severity, and distress associated with, 32 separate, multidimensional symptoms experienced by patients. - RECIST v.1.1 criteria: to assess tumor response - Earle criteria: to assess the aggressiveness of health care		

*CCI, Charlson Comorbidity Index; CEA, Carcinoembryonic Antigen; CYFRA 21-1, Cytokeratin 19 fragment; ERCC1, Excision Repair Cross-Complementation Group 1; HADS, Hospital Anxiety and Depression Scale; MSAS-SF, Memorial Symptom Assessment Scale-Short Form; PD-L1, Programmed Death-Ligand 1; RRM1, Regulatory Subunit of Ribonucleotide Reductase 1; SF-36, Short-Form 36*.

### Nominal Group Meetings

The aim of the nominal groups was to reach consensus on the variables to ultimately be included in the standard set of health outcomes for LC in Spain as well as to define the measurement instrument and frequency of measurement to be used.

Nominal group is a qualitative methodology which allows to achieve consensus, while assuring a balanced participation among group members that are given equal opportunity to share their opinion (17). The methodology involves five steps tailored to meet the purpose of the meeting as follows (18): (1) Introduction and explanation: welcoming and explanation of the purpose and procedure of the meeting; (2) Silent generation of ideas: each participant evaluated individually (without consulting or discussing with others) the variables proposed; (3) Sharing ideas: participants shared individually the variables they had selected; (4) Group discussion: participants could seek verbal explanation or further details about any of the variables that other participants had proposed; (5) Voting and ranking: during this phase, participants were asked to prioritize the variables proposed. Variables were included if ≥75% of participants agreed on their inclusion.

In order to be representative at a national level, four nominal groups were conducted, involving experts from different Spanish geographic areas. In each nominal group, participation was limited to a maximum of 12 experts, including oncologists, hospital pharmacists, hospital managers, and patient representatives. Participants of the nominal groups were identified by the scientific committee, in collaboration with patient advocacy groups (*Asociación Española de afectados de Cáncer de Pulmón* [AEACaP] and *Grupo Español de Pacientes con Cáncer [GEPAC]*), and the study coordinator. The selection was based on the participant experience in LC management, PRO measurement as well as scientific contributions, availability, and interest in the project.

### Second Scientific Committee Meeting

A group discussion was carried out to review the results obtained during the four nominal groups, and to determine the inclusion/exclusion of those variables for which a consensus was not reached during the meetings. Consensus was reached if ≥75% of the members of the scientific committee agreed on the variable inclusion/exclusion. Based on the meeting results, the variables to be included in the standard set of health outcomes for LC were defined.

## Results

### Literature Review

**Figure 1:**
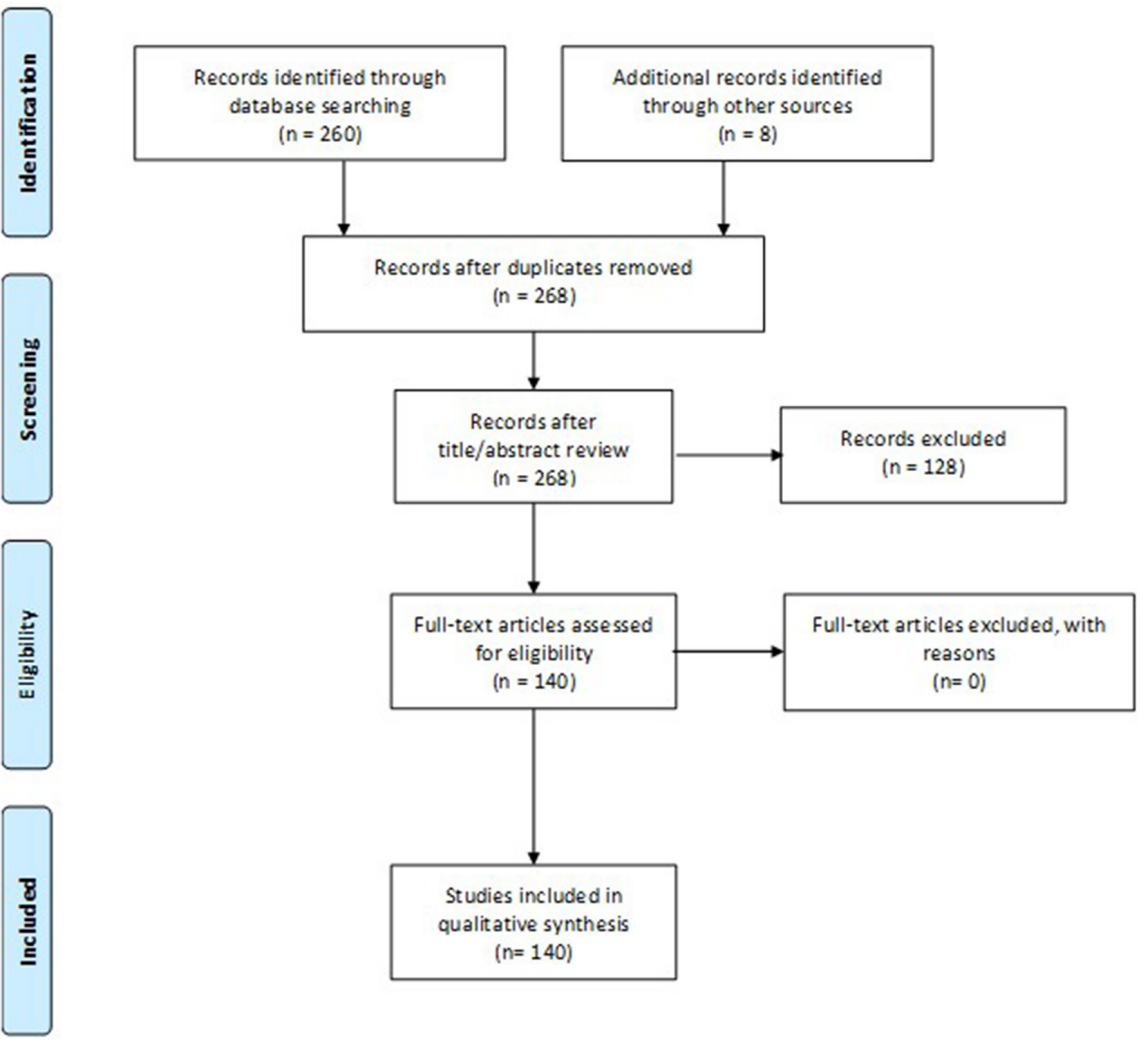
PRISMA Flow diagram.

The database search yielded 260 references, while 8 additional publications were identified by hand-searching. A total of 128 publications were excluded by title and abstract. The full texts of the remaining 140 articles were assessed for eligibility. All of them were included in the qualitative synthesis and reviewed to identify variables, and the respective measuring instruments, that might have not been identified in the review conducted by ICHOM. A flow diagram of the selection process according to the PRISMA Guidelines is depicted in Figure 1.

Following publication screening, a total of 14 variables (6 case-mix and 8 outcomes variables) and 6 measuring instruments, that had not been included in the ICHOM catalog, were identified (Table 2).

### First Scientific Committee Meeting

Through the review of the variables included in the ICHOM standard set (Table 1) as well as those identified through the updated literature review (Table 2), a total of 17 case-mix and 25 outcomes variables were selected by the scientific committee to be presented and evaluated during the nominal groups. Selected variables, together with their measuring instruments are presented in Supplementary Tables 2, 3. During the meeting, the scope of the standard set was also defined. In line with the ICHOM standard set for LC, the target population of the standard set defined within the present project framework was set to include all patients with newly diagnosed LC, regardless of the disease stage, type or therapeutic options.

### Nominal Group Meetings

Overall, 13 oncologists, 14 hospital pharmacists, 4 hospital managers and 3 LC patients participated in the nominal group meetings. Thirty-five case-mix variables and 45 outcomes variables were proposed during the meetings. Nevertheless, consensus was only achieved for 13 case-mix (n=2 demographic factors, *n* = 3 baseline clinical factors, n = 7 baseline tumor factors and n = 1 treatment factors) and six outcomes variables across the four nominal groups (Supplementary Tables 2, 3).

### Second Scientific Committee Meeting

Based on the results achieved during the nominal group meetings, the scientific committee identified the variables to be included in the standardized set of health outcomes for LC. The variables finally included in the standard set are presented below.

#### Case-Mix Variables (Table 3)

Case-mix variables include those that allow patient characterization. Assessment of these variables should be carried out in the first visit after diagnosis, prior to treatment initiation.

**Table 3:** Spanish standard set of patient-centered outcomes in LC.

**Patient profile**	**Variable**	**Supporting information**	**Measurement instrument**	**Timing**	**Data sources**
**Demographic factors**					
All patients	Age		Date of birth	Baseline (before treatment begins)	Clinical
	Gender		F: female; M: male		Clinical
	Family support	Degree of family support and degree of patient dependence	Yes/No		Patient-reported
	Educational level	Level of schooling completed	(0) Without studies; (1) primary school level; (2) secondary school level; (3) higher education		Patient-reported
**Baseline clinical factors**					
All patients	Unintentional weight loss		Yes/No/I don't know	Baseline (before treatment begins)	Patient-reported
	Smoking status	Smoking status at diagnosis	Pack-year index + smoking status classification: Never-smoker (<100 cigarettes in lifetime), ex-smoker (stopped >1 year before diagnosis), current smoker		Patient-reported
	Performance status		ECOG scale		Clinical
	Patient-reported health status		Tracked via generic questionnaire EQ5D and LC specific questionnaire LCSS		Patient-reported
	Comorbidities		Charlson index		Clinical
	Pulmonary function	FEV_1_	NA		Clinical
**Baseline tumor factors**					
All patients	Clinical stage		TNM scale	Baseline (before treatment begin)	Clinical
	Pathological stage		TNM scale		Clinical
	Histology		NA		Clinical
	EGFR mutation^*^		Yes/No/undetermined		Clinical
	ALK translocation^*^		Yes/No/undetermined		Clinical
	ROS-1 rearrangement ^*^		Yes/No/undetermined		Clinical
	PD-L1 expression^*^		Yes/No/undetermined		Clinical
**Treatment factors**					
All patients	Treatment intent		(1) curative; (2) palliative	Baseline (before treatment begins)	Physician-reported
	Completed treatment		(1) Yes, with dose reduction; (2) No, due to toxicity; (3) No, due to patient's will; (4) No, due to patient's death	At treatment ending	Physician-reported

*Case-mix variables*.

NA, not applicable; ECOG, Eastern Cooperative Oncology Group; EQ-5D, EuroQol; LCSS, Lung Cancer Symptoms Scale; FEV-1, forced expiratory volume; EGFR, Epidermal growth Factor Receptor; ALK, Anaplastic Lymphoma Kinase; PD-L1, Programmed Death-ligand;

* *List of Biomarkers annually valuated and updated*.

In addition to the sociodemographic variables included in the original ICHOM standard set (age and gender), it was agreed to collect the family support, as it may reflect the patient's family environment, as well as, the degree of patient dependence. Even though consensus was not reached among the nominal groups about whether to include educational level as a case mix variable, the scientific committee considered appropriate to include it as a surrogate measurement of socioeconomic status. This decision was motivated by previous studies showing a relationship between educational level and patient prognosis (19, 20). Conversely, despite ICHOM recommendation, collection of ethnicity data was considered not relevant to the Spanish setting, and was, therefore, excluded from the set.

Aligned with the ICHOM recommendations, it was agreed to gather data regarding the following baseline clinical factors: unintentional weight loss, smoking status, comorbidities, performance status and patient's HRQoL. In addition, due to its prognostic value, assessment of pulmonary function, through the measurement of forced expiratory volume (FEV-1), was also included in the set.

Consensus was reached on the use of Charlson Comorbidity Index (21) to collect the comorbidities. Experts discouraged the use of the questionnaire suggested by ICHOM, the Modified Self-administered Comorbidity Questionnaire (22) for two main reasons. First, a Spanish translation and validation of the questionnaire is not currently available. Second, experts considered that self-reporting of comorbidities may lead to underestimating of the comorbidities perceived by patients as irrelevant or unknown.

To assess patients' performance status, experts agreed on the use of the Eastern Cooperative Oncology Group (ECOG) scale. This scale describes a patient's level of functioning in terms of self-care, carrying out daily activities, and physical ability (23).

The ICHOM proposed instruments to measure patient-reported health status [EORTC-QLQ-C30 (24) and the LC specific version QLQ-LC13 (25)] were not deemed appropriate in the Spanish setting, as they are burdensome to complete and their use is limited in routine clinical practice. Instead, consensus was reached to use the generic questionnaire EuroQol EQ-5D (26, 27), that is currently widely used in the Spanish setting, and the LC specific questionnaire Lung Cancer Symptom Scale (LCSS) (28), for which a Spanish validated version is already available. The combined use of both questionnaires allows to gather information about patient perspective on their physical and emotional functioning, fatigue, vitality, pain, cough, breathing difficulty, hemoptysis, and loss of appetite.

The clinical and pathological stage, defined by the TNM staging scale, tumor histology and basis of diagnosis (clinical, histological, or cytological), were identified as key variables to be recorded as a case-mix variable.

Consensus was reached on including LC predictive biomarkers such as EGFR gene mutational status, ALK gene translocation, ROS-1 rearrangement and PD-L1 expression. Due to the continuous advances on the molecular mechanisms of LC, it was agreed to annually evaluate and update the list of biomarkers to be included in the standard set.

Regarding patient treatment, it was agreed to collect data on treatment intention (curative vs. palliative) and whether treatment was completed (with or without dose reduction) or non-completed (due to toxicity, patient's decision or patient's death).

#### Outcomes (Table 4)

##### Survival

LC is associated with high mortality rates (1). Thus, overall survival was considered a key variable to be included in the standard set for patient's follow up. Moreover, participants agreed on gathering information regarding the cause of death, indicating whether it was tumor or treatment related. Conversely, following ICHOM recommendations, progression-free survival (PFS) was excluded from the standard set. Although PFS is commonly assessed in most clinical trials as a measure of disease control, it was considered potentially unreliable due to ascertainment bias and, ultimately, less important than overall survival.

**Table 4:** Spanish standard set of patient-centered outcomes in LC.

**Patient profile**	**Measure**	**Supporting information**	**Measurement instrument**	**Timing**	**Data sources**
**Degree of Health**					
All patients	Performance status		ECOG scale	During follow-up visits	Clinical
	Patient-reported health status	Global health status, physical and emotional function	Tracked via generic questionnaire EQ5D and LC specific questionnaire LCSS	At 3, 6, and 12 months. Later, tracked annually for life^*^	Patient-reported
		Fatigue, vitality, pain, cough, difficulty breathing, hemoptysis and loss of appetite	Tracked via LC specific questionnaire LCSS		
**Survival**					
All patients	Overall survival		Date of death	NA	Administrative data (death registry)
	Cause of death	Tumor/treatment related or not	NA		Clinical
**Quality of death**					
All patients	Place of death		NA	Date of death	Administrative data (death registry)
	Aggressive intervention and palliative care	Earle criteria: (1) patient receives chemotherapy or other antineoplastic therapy in last 14 days of life; (2) patient initiates a new therapeutic scheme in the last month of life; (3) patient goes to emergency room more than once in the last month of life or ICU admitted; (4) patient dies at an oncology unit instead of receiving palliative care; (5) patient does not receive palliative care before passing away; (6) patient dies while receiving palliative care in the last 72 h before hospital admission		30 days before death	Clinical
	Existence or doctor's knowledge about the living will of patients		Yes/No	NA	Patient-reported
**Acute complications of treatment**					
Patient receiving surgical resection	Major surgical complications	(1) Secondary complication related to surgical care; (2) Urgent re-admission after the next 7 days post-discharge, for a cause related to surgical treatment; (3) Death after surgery (in the next 30 days)		NA	Clinical
Patient with systemic therapy or/and radiotherapy	Major systemic therapy or/and radiotherapy complications	Presence of grade ≥3	CTCAE^TM^	NA	Clinical
			PRO- CTCAE^TM^	NA	Patient reported
**Others**					
All patients	Time from diagnosis		Date of hospital admission or non-hospital consultation when data of the histological or cytological confirmation is unknown	At diagnosis	Clinical
	Time from diagnosis to treatment		NA	When treatment begins	Clinical
	Patient productivity loss	Sick leave or disability	Yes/No	NA	Patient reported

*Outcomes variables*.

NA, not applicable; ECOG, Eastern Cooperative Oncology Group; EQ-5D, EuroQol; LCSS, Lung Cancer Symptoms Scale;

* *when treatment is change, Patient-reported health status will be evaluated at 3, 6 and 12 months; CTCAE, Common Terminology Criteria for Adverse events; PRO-CTCAE, Patient-Reported Outcomes version of the CTCAE*.

##### Complications of treatment

In line with ICHOM classification, treatment complications were divided to surgery-related or associated with systemic therapy and/or radiotherapy. Participants agreed on monitoring surgery-related complications using the *Observatorio de Resultados del Servicio Madrileño de Salud* (Outcomes Observatory of Madrid Healthcare Services) classification. This classification includes the reporting of whether the patient: (1) suffers a secondary complication related to surgical care, (2) requires an urgent re-admission for a cause related to surgical treatment in the first 7 days following discharge, or (3) dies in the first 30 days after surgery (29).

In regard to the complications of systemic therapy and/or radiotherapy, participants emphasized the importance of both patient self-reporting and clinician reporting of adverse events. Therefore, a consensus was reached to collect all grade 3 or higher adverse events using both the Common Terminology Criteria for Adverse Events (CTCAE) and Patient-Reported Outcomes version of the CTCAE (PRO-CTCAE). Although the use of such instruments might be time-consuming, participants agreed that a standardized collection of treatment toxicities is necessary to avoid the risk of overlooking important complications, and to ensure their inclusion in the medical record.

##### Degree of health

HRQoL and performance status are frequently impaired in LC patients (5, 8). Therefore, and in line with ICHOM recommendations, it was agreed to collect both variables at baseline and during patients' follow up. As previously indicated, ECOG scale (23) was selected to assess patients' performance status, while the EQ-5D (26, 27) and LCSS questionnaire (28) were chosen for the assessment of patients' HRQoL.

##### Quality of death (end of life care)

Ensuring an adequate quality of end-of-life care as well as the respect of patients' wills has become a key healthcare objective (30). Therefore, besides capturing place of death, participants stressed the importance of assessing the alignment between the care received at the end of life and patients' living will. For this purpose, it was agreed to register whether patients had a living will, received aggressive care, and had access to palliative care in the last 30 days of life, using Earle Criteria adapted for individual patient use (Table 4). Earle Criteria evaluate quality of end of life care by assessing: (a) administration of new anticancer therapies or continuation of ongoing treatments very near death, (b) number of emergency room visits, inpatient hospital admissions or intensive care unit days near the end of life and (c) inadequate access to palliative care and/or enrollment in hospice (31).

##### Others

Experts agreed to report both the date of diagnosis and the time interval from diagnosis to treatment. Patient's and/or caregiver's working life might be substantially influenced by both the disease and treatment, causing potential situations of absenteeism, presentism, or sick leave. In this light, experts agreed to report patient's and/or caregiver's productivity loss, by collecting sick leave or occupational disability for both patients and caregivers.

## Discussion

In clinical practice, measurement of outcomes that matter to LC patients, aside from survival, remains limited. However, to promote patient-centered care, the collection of outcomes based on the patient's priorities is crucial. Experience from the implementation of ICHOM standard sets in clinical practice, in diseases such as hip and knee osteoarthritis (32), cleft lip and palate (33), coronary artery disease (34) or Parkinson's Disease (35), has shown a positive impact on all phases of the care process. Likewise, a one-year pilot study conducted in Netherlands demonstrated that the ICHOM standard set can be implemented during routine lung cancer treatment without significantly disturbing the routine workflow (36, 37). The authors concluded that the collection of PROs is not too time-consuming, however it requires *ad hoc* tools and dedicated staff (36, 37).

Patient information collated via PRO measures (PROMs) allows clinicians to explore the perspective of the patients on different aspects of the disease, during the follow-up.

This, in turn, promote a better involvement of the patient in disease management (32–35). Moreover, PROMs use provides the opportunity for patients to engage in their consultation in advance, increasing self-awareness of their health and helping them to tailor the consultation to their needs (32–35). Of note, healthcare professionals also perceive the use of these standard sets as a valuable tool that facilitates benchmarking. Indeed, clinicians can learn from the outcomes data they gather and, at the same time, share their knowledge and learn from the experience of other healthcare professionals in different settings (32–35).

Adaptation of the ICHOM standard set for LC to the Spanish setting are essential steps in order to allow its implementation in routine clinical practice since it allows to identify those variables and its instruments, proposed by ICHOM that (1) are routinely collected in Spanish clinical practice and/or Spanish clinicians and patients are familiarized with; (2) the technology needed to measure them is available in the Spanish setting; and (3) the instruments to collect them are available and validated in Spanish language.

In this regard, several of the PROMs proposed by ICHOM have either not yet been validated or have had limited use in the Spanish context, thus hindering their use in daily practice. Thus, although divergence from the original standard set developed by ICHOM might partly complicate international benchmarking of LC patients' outcomes, selecting the most suitable PROMs to our setting is essential to ensure their use.

This standard set reflects the opinion of a group of 35 experts on the management of LC and 5 patient representatives. Since one of the main purposes of the standard set is to reflect outcomes that matter to patients, their broad participation in the project is one of its main strengths. Patient representatives have been actively involved in the decision-making process as members of the scientific committee or participants in the nominal groups. Although no major differences are expected, it is important to highlight that different groups of experts and patients could have agreed on different recommendations. In order to minimize this potential bias, and ensure national representativeness, participants from four broad geographic areas were involved in the project.

It must be acknowledged that the update of the literature review, on which the present standard set is based, covers studies published until 31/12/2017. Thus, some relevant variables (e.g., the determination of recently identified LC related biomarkers) may have not been considered when elaborating the standard set. To minimize this limitation, and due to the continuing advances in both the knowledge and treatment of this disease, we recommend to periodically update the list of biomarkers to evaluate during patient follow-up. In addition, we also suggest that the present standard set be periodically updated.

Of note, the present adaptation of the ICHOM standard set for LC represents a starting point, and several barriers need to be addressed on the road to its successful implementation in the Spanish setting. Namely, the time required for the collection of the variables proposed, the lack of digital tools allowing a systematic and automatic PROMs compilation, together with a limited education and information of patients and clinicians about PROMs, have been identified as the main barriers to the implementation of the present standard set (38). Newer platforms for data collection, based on information and communication technologies, may reduce both patient and clinician burden, as well as, data processing time, thus facilitating the use or PROMS in clinical practice (39). Other barriers to be tackled to ensure widespread use of this standard set are inherent to the structure of the Spanish national healthcare system (SNHS). Indeed, one of the main characteristics of the SNHS is its heterogeneity: healthcare processes, organizational models as well as information systems differ widely both among and within regions. This heterogeneity has been also observed in the context of LC, with a recent study showing remarkable differences among Spanish regions in the provision of care for LC patients (40). Overall, these data indicate the need to promote harmonization of the best practice in LC management and treatment to guarantee the success of this initiative.

Besides addressing the aforementioned barriers, a further step to test the feasibility and promote the integration of the defined standard set into the Spanish healthcare model may involve the conduction of a pilot implementation study. Indeed, due to the heterogeneity of SNHS, a pilot study is key to determine the feasibility and viability of introducing the standard set in routine clinical practice. The results of the pilot study may provide insights into the main resource requirements and organizational challenges to be tackled during the implementation.

Although data from Standard Set implementation is limited, previous experiences suggest that several requirements are needed to ensure its implementation in a particular setting: (1) appropriate staff to support the implementation of PROMs tools, facilitate ongoing data collection, and maximize the completeness of data collection and, (2) adequate technology to support for database/platform development. Moreover, during the implementation process the knowledge of existing workflows for patient assessment and review, and the patient's journey in the hospital system is crucial.

## Conclusion

The present adaptation of ICHOM standard set may facilitate its implementation in Spanish clinical practice, paving the way to standardize the collection of variables in LC, and promoting the incorporation of patients' perspective in LC management. In turn, the information provided through the systematic compilation of this set of variables may allow both clinicians and health policy makers to define strategies aimed at achieving high-quality and patient-centered care.

## Data Availability Statement

All datasets generated for this study are included in the article/Supplementary Material.

## Ethics Statement

Ethical review and approval was not required for the study on human participants in accordance with the local legislation and institutional requirements.

## Author Contributions

VE-V, AC, MB, RC-B, CP, CG, JS, AO, and LL contributed to the conceptualization and design of the study. MA contributed to the conceptualization and design of the study, data curation, and data analysis. MC contributed to the conceptualization and design of the study, data curation, data analysis, and wrote the first draft of the manuscript. All authors contributed to manuscript revision, read, and approved the submitted version.

## Conflict of Interest

VE-V has received support to continuing education/advisory fees from Amgen, Astellas, AstraZeneca, Boehringer-Ingelheim, Bristol-Myers Squibb, Ipsen Pharma, Janssen, and Merck Sharp & Dohme. AC has received honorary/consulting fees from AstraZeneca, Boehringer-Ingelheim, Pfizer, Roche/Genentech, Eli Lilly and Company, Novartis, Merck Sharp & Dohme, and Bristol-Myers Squibb. MB has received support to continuing education/advisory fees from Abbvie and Roche. RC-B has received support to continuing education/advisory fees: Boehringer-Ingelheim, Janssen and Merck Sharp & Dohme, Hoffmann-La Roche and Pfizer. CP, CG, JS, and AO are employees of Bristol-Myers Squibb. MA, MC, and LL work for an independent research entity that received funding from Bristol-Myers Squibb to coordinate and conduct the study.
