# Supplementary material for: Standardizing Health Outcomes for Lung Cancer. Adaptation of the International Consortium for Health Outcomes Measurement Set to the Spanish Setting
Source: Front Oncol. 2020 Sep 2;10:1645. doi: 10.3389/fonc.2020.01645 (PMC7492557; doi:10.3389/fonc.2020.01645)
Supplement: Supplementary file 1 [file Data_Sheet_1.docx]

Supplementary Material

# Supplementary Tables

Supplementary table 1. Literature review characteristics.

| Database | Pubmed/Medline |
| --- | --- |
| Search Period | 01/01/2015- 31/12/2017 |
| Search Strategy | ("lung neoplasms"[MeSH Terms] AND (randomized controlled trial[All Fields] OR randomized controlled trials[All Fields] OR randomized controlled trial[All Fields] OR randomised controlled trials[All Fields] OR randomized controlled trial[Publication Type] OR Review[ptyp] OR systematic[sb]) AND ("Quality of Life"[Mesh] OR "Quality Indicators, Health Care"[Mesh] OR "Patient Outcome Assessment"[Mesh] OR patient reported outcome*[tiab] OR patient related outcome*[tiab] OR patient-reported outcome*[tiab] OR patient-related outcome*[tiab] OR patient reported outcome*[ot] OR patient related outcome*[ot] OR patient-reported outcome*[ot] OR patient-related outcome*[ot] OR "Treatment Outcome"[Mesh]) |
| Filters | English [Lang]; Clinical Trials |

Supplementary table 2. Case mix variables proposed during the scientific committee and nominal group meetings.

Legend:

ALK: Anaplastic lymphoma kinase; BMI: body mass index; BRAF: v-raf murine sarcoma viral oncogene homolog B1; EGFR: Epidermal Growth Factor Receptor; FEV-1: forced expiratory volume; CV: Cardiovascular; PD-L1: Programmed Death-ligand 1; ROS-1: Reactive Oxygen Species.

| **Scientific Committee meeting** | **Nominal group 1** | **Nominal group 2** | **Nominal group 3** | **Nominal group 4** |
| --- | --- | --- | --- | --- |
| **Demographic factors** | | | | |
| - Age - Gender - Ethnicity - Educational level | - Age - Gender - Ethnicity - Height and weight - Family status - Occupational exposure | - Age - Gender - Ethnicity - Family support - Employment status | - Age - Gender - Ethnicity - Educational level - Occupational exposure | - Age - Gender - BMI - Occupational exposure to asbestos - Employment status - Socio-economic status - Presence of a caregiver |
| **Baseline clinical factors** | | | | |
| - Unintentional weight loss - Smoking status (pack-year index) - Pulmonary function (FEV_1_) - Comorbidities (Modified Self-administered Comorbidity Questionnaire) | - Unintentional weight loss - Smoking status (pack-year index) - Pulmonary function (FEV_1_) - Comorbidities (according to clinical record: CV disease, pulmonary, renal or liver disease, endocrine disorders, immunological disease, recent surgery) | - Unintentional weight loss - Smoking status - Pulmonary function (FEV_1_)   Comorbidities (according to clinical record: CV disease, pulmonary, renal or liver disease, auto-immune disease, diabetes, recent surgery, hearing impairment). | - Unintentional weight loss - Smoking status (pack-year index) - Comorbidities (Modified Self-administered Comorbidity Questionnaire) - Health-related quality of life (any validated instrument) | - Unintentional weight loss - Smoking status (pack-year index + classification as smoker, non-smoker, ex-smoker) - Comorbidities |
| Baseline tumor factors | | | | |
| - Clinical stage (TNM) - Pathological stage (TNM) - Histology - EGFR mutation - ALK translocation - ROS-1 rearrangement - PD-L1 expression | - Clinical stage (TNM) - Pathological stage (TNM) - Histology - EGFR mutation - ALK translocation - ROS-1 rearrangement - PD-L1 expression | - Clinical stage (TNM) - Pathological stage (TNM) - Histology - EGFR mutation - ALK translocation - ROS-1 rearrangement - PD-L1 expression - BRAF - % of diagnosis based on biopsy/cytology | - Clinical stage (TNM) - Pathological stage (TNM) - Histology - EGFR mutation - ALK translocation - ROS-1 rearrangement - PD-L1 expression | - Clinical stage (TNM) - Pathological stage (TNM) - Histology - EGFR mutation - ALK translocation - ROS-1 rearrangement - PD-L1 expression - BRAF - Number of metastases - Brain metastases |
| **Treatment Factors** | | | | |
| - Treatment intent (curative/palliative) - Completed treatment (w/o dose reduction) | - Treatment intent (curative/palliative) - Completed treatment (w/o dose reduction) - Allergies - Treatment withdrawal (with reasons) | - Treatment intent (curative/palliative) - Completed treatment - Treatment withdrawal - Dose reduction | - Treatment intent (curative/palliative) | - Treatment intent (curative/palliative) - Completed treatment (w/o dose reduction) - First line of treatment - Administration route (oral, intravenous, subcutaneous) - Local treatment received (type) |

Supplementary table 3. Outcomes variables proposed during the scientific committee and nominal group meetings.

Legend:

CTCAE: Common Terminology Criteria for Adverse events; ECOG: Eastern Cooperative Oncology Group; EORTC: European Organisation for Research and Treatment of Cancer; EQ-5D: EuroQol; ER: emergency room; HRQoL: Health related quality of life; LCSS: Lung Cancer Symptoms Scale; PRO-CTCAE: Patient-Reported Outcomes version of the CTCAE; QLQ- LC13:Lung Cancer-specific quality of life questionnaire; SERMAS: Servicio Madrileño de Salud

| **Scientific Committee meeting** | **Nominal group 1** | **Nominal group 2** | **Nominal group 3** | **Nominal group 4** |
| --- | --- | --- | --- | --- |
| **Degree of health** | | | | |
| - Performance status (ECOG) - Patient-reported health status: Global health status, physical and emotional function; fatigue, vitality, pain, cough, difficulty breathing, hemoptysis, loss of appetite, insomnia, constipation, diarrhea, weight loss (measured with a specific and a general HRQoL questionnaire) | - Performance status (ECOG) - Patient-reported health status: Global health status, physical and emotional function; fatigue, vitality, pain, cough, difficulty breathing, hemoptysis, loss of appetite, (measured with LCSS + EQ-5D) | - Performance status (ECOG) - Patient-reported health status: Global health status, physical and emotional function; fatigue, vitality, pain, cough, difficulty breathing, hemoptysis, loss of appetite, (measured with LCSS + EQ-5D) or Global health status, physical and emotional function; pain, cough, difficulty breathing, hemoptysis, weight loss, (measured with EORT-QLQ-LC-13 + EQ-5D) | - Performance status (ECOG) | - Performance status (ECOG) - Patient-reported health status: Global health status, physical and emotional function; fatigue, vitality, pain, cough, difficulty breathing, hemoptysis, loss of appetite, (measured with LCSS + EQ-5D) |
| **Survival** | | | | |
| - Overall survival - Cause of death | - Overall survival - Cause of death - Number of treatment lines - Progression-free survival - Progression-free survival (1^st^ treatment line) | - Overall survival - Cause of death | - Overall survival - Cause of death - Number of treatment lines - Progression-free survival (clinical evaluation and/or radiographic progression) | - Overall survival - Cause of death - Progression-free survival - Clinical benefit (time) - Time to treatment failure - Time to next treatment |
| **Quality of death** | | | | |
| - Place of death - Length of hospital stay during end of life period - Aggressive intervention and palliative care (Earle criteria) - Existence or doctor´s knowledge about the living will of patients | - Place of death - Length of hospital stay during end of life period - Aggressive intervention and palliative care (Earle criteria 1, 2, 5 and 6) - Doctor´s knowledge about the living will of patients - Access to palliative care (y/n) | - Place of death - Aggressive intervention and palliative care (Earle criteria 1-3) - Existence or doctor´s knowledge about the living will of patients | - Place of death - Therapeutic aggressiveness in the end of life (antineoplastic treatment in the last 14 days) and access to palliative care (at least 3 months before death). - Existence or doctor´s knowledge about the living will of patients | - Place of death - Length of hospital stay during end of life period - Administration of active treatment in the last month (y/n) - Change of treatment in the last month (y/n) - ICU admission in the last month (y/n) - ER admission in the last month - Access to palliative care in the last month |
| **Acute complications of treatment** | | | | |
| - Major surgical complications (Indicators of the SERMAS observatory) - Major systemic therapy or/and radiotherapy complications (CTCAE and PRO-CTCAE) | - Major surgical complications (surgical complications and post-operative mortality) - Major systemic therapy or/and radiotherapy complications (CTCAE and PRO-CTCAE) | - Major surgical complications (surgical complications and post-operative mortality) - Major systemic therapy or/and radiotherapy complications (CTCAE and PRO-CTCAE) | - Major surgical complications (Indicators of the SERMAS observatory) - Major systemic therapy or/and radiotherapy complications (CTCAE and PRO-CTCAE) | - Major surgical complications (surgical complications, re-admission due to surgical complications and post-operative mortality) - Major systemic therapy or/and radiotherapy complications (list of the 5 most relevant complications according to treatment type) - Treatment withdrawal due to toxicity - Patient wish to withdraw the treatment due to toxicity |
| **Others** | | | | |
| - Date of diagnosis - Productivity loss of the patient and caregiver (sick leave) - resource use (ER admission, unscheduled specialist visits, hospitalizations) | - Date of diagnosis - Productivity loss of the patient and caregiver (sick leave) - resource use (ER admission, unscheduled specialist visits, hospitalizations) - Treatment start date - Patients’ preferences | - Date of diagnosis - Productivity loss of the patient and caregiver (sick leave) - resource use (ER admission, unscheduled specialist visits, hospitalizations, nurse visit) - Treatment start date | - Date of diagnosis - Independence in activities   of daily living | - Date of diagnosis - Adherence to treatment - Time to diagnosis - Time to first treatment |
